# A Novel HILIC‐UPLC‐DAD‐MS/MS Method for the Analysis of Mycosporine‐Like Amino Acids and Their Quantification in Diverse Algae

**DOI:** 10.1002/elps.70110

**Published:** 2026-05-23

**Authors:** Armin Oberosler, Anastasiia Fedorova, Ignacio Zegri, Fabian Hammerle, Michael Zwerger, Thomas Werner, Markus Ganzera

**Affiliations:** ^1^ Department of Pharmacognosy University of Innsbruck Innsbruck Austria; ^2^ Department of Chemistry University of Paderborn Paderborn Germany; ^3^ Institute of Analytical Chemistry and Radiochemistry University of Innsbruck Innsbruck Austria

**Keywords:** hydrophilic interaction liquid chromatography (HILIC), marine organisms, mycosporine‐like amino acids (MAAs), ultra‐performance liquid chromatography (UPLC)

## Abstract

Mycosporine‐like amino acids (MAAs) are photoprotective compounds primarily produced by marine organisms, especially red algae. Chemically, MAAs and their precursors are low‐molecular‐weight natural products that absorb ultraviolet radiation in the range of 270–360 nm without generating free radicals. Owing to these properties, MAAs have attracted considerable interest for potential pharmaceutical and cosmetic applications as natural sunscreen agents. In this study, the first hydrophilic interaction liquid chromatography‐ultra‐performance liquid chromatography (HILIC‐UPLC) method, coupled with both diode array detector (DAD) and tandem mass spectrometry (MS/MS), was developed for the determination of 11 MAAs and 2 MAA precursors in various algal species. As column, a YMC‐Triart Diol‐HILIC (particle size: 1.9 µm) was used, and the separation of the MAAs realized in under 5 min. The validation of the method was carried out following International Council for Harmonisation (ICH) guidelines, demonstrating linearity, selectivity, precision, and accuracy. The analysis of extracts prepared from well‐known species, such as *Porphyra* sp. and *Chondrus crispus*, confirmed practical applicability. Demonstrating feasibility of MS/MS‐based quantification of co‐eluting compounds, the method represents an appealing alternative that surpasses established procedures not only in terms of analysis time but also by providing a substantially increased informative value.

## Graphical abstract

The separation of selected mycosporine‐like amino acids and two biosynthetic precursors, in total 13 compounds, by HILIC‐UPLC is described for the first time. With an analysis time of only 5 min, it surpasses all currently known methods for analysis of these promising natural products.

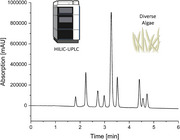

**Abbreviations**
DW: dry weight
MAAs: mycosporine‐like amino acids
UPLC: ultra‐performance liquid chromatography

## 1 Introduction

Mycosporine‐like amino acids (MAAs) are small, water‐soluble metabolites with strong UV‐absorbance that occur widely in marine and freshwater organisms, including cyanobacteria, algae, and invertebrates [1, 2]. Their potential as natural photoprotective agents has stimulated commercial interest, exemplified by algal extracts incorporated into cosmetic products, such as Helioguard 365 and Helionori [3, 4]. Structurally, MAAs are characterized by a cyclohexenone or cyclohexenimine chromophore, usually conjugated to amino acids, amino alcohols, or related substituents [1]. The conjugated double bonds within the ring system are responsible for their characteristic absorption maxima between approximately 270 and 360 nm, whereas their high molar extinction coefficients [5], typically up to 50 000 M^−1^×cm^−1^, underline their efficiency as UV filters [1, 6]. The physicochemical ability of MAAs to absorb UV light and convert energy into heat without the production of free radicals is referred to as “button on a string” mechanism [7].

Since the first MAA was described in the mid‐20th century, followed by the isolation of well‐known representatives like shinorine thereafter [8], analytical techniques for their detection and separation have advanced considerably. Early studies relied on thin‐layer chromatography (TLC), which offered only limited resolution [9]. The use of high‐performance liquid chromatography (HPLC) enabled more accurate quantification and improved separation of structurally related MAAs [10]. More recently, hydrophilic interaction liquid chromatography (HILIC) has also been described for the separation of these highly polar compounds, providing excellent retention and separation efficiency. Hartmann et al. developed such a method for the identification of five MAAs. However, quantification was only achieved for porphyra‐334, shinorine, and palythine. Furthermore, a single analysis using this HILIC method required approximately 30 min, not including 15 min for re‐equilibration [11]. Another validated HILIC method was reported in 2019 for the separation of two MAAs, mycosporine‐glutaminol and mycosporine‐glutamicol in 16 min [12]. In the last decade, the introduction of U(H)PLC has offered further improvements in terms of resolution and speed [13, 14]. For instance, rather recently a UHPLC‐diode array detector (DAD) method has been reported to separate more than ten MAAs within 8 min analysis time, achieving excellent resolution and reproducibility [15]. Moreover, tandem mass spectrometry (MS/MS)‐based detection techniques have been introduced in the area of MAA research, however, only accounting for the three MAAs shinorine, porphyra‐334, and mycosporine‐glycine‐alanine [16, 17].

On the basis of these interesting analytical options, the current study investigates whether HILIC‐ultra‐performance liquid chromatography can be applied for MAA analyses as well, in order to further reduce separation time while maintaining or improving resolution. Target analytes included the two biosynthetic MAA precursors 4‐deoxygadusol (**1**) and gadusol (**3**), along with 11 MAAs, which are, mycosporine‐serinol (**2**), usujirene (**4**), palythene (**5**), aplysiapalythine B (**6**), mycosporine‐methylamine‐threonine (**7**), palythine (**8**), aplysiapalythine A (**9**), asterina‐330 (**10**), mycosporine‐alanine‐glycine (**11**), porphyra‐334 (**12**), and shinorine (**13**). Regardless to say that the developed method should also fulfill validation criteria in accordance with internationally recognized guidelines (i.e., International Council for Harmonisation [ICH]) [18].

## 2 Materials and Methods

### 2.1 Biomaterial and Preparation of Crude Extracts

Different nori (*Porphyra* sp.) samples were obtained from local supermarkets in Innsbruck in 2025; other specimens analyzed were available in‐house from previous projects. They were collected in 2018 in Brittany (France) and morphologically identified by Prof. Dr. U. Karsten from the University of Rostock, Germany; voucher specimens of all samples are deposited at the Department of Pharmacognosy, University of Innsbruck, Austria. Sample preparation and extraction followed the protocol of Zwerger and Ganzera [15] with one slight modification. Briefly, 50 mg of biomaterial was extracted three times with 5 mL of ultrapure water using an ultrasonic bath (Bandelin SONOREX, Berlin, Germany) for 15 min at room temperature. The extracts were centrifuged for 5 min at 1500 × *g*, the clear supernatants combined and diluted to 25 mL with water in a volumetric flask. An aliquot (1 mL) was evaporated under a gentle stream of air and reconstituted in 500 µL of acetonitrile/water (ACN/water) (9:1, v/v). Completeness of extraction was verified by extracting one sample (*Vertebrata lanosa*) a fourth time. Analyzing it for possibly remaining MAAs revealed that the respective levels were below the limit of detection (LOD).

### 2.2 Chemicals and Reagents

ACN was purchased from Merck (Darmstadt, Germany), ammonium acetate from Serva (Heidelberg, Germany), and acetic acid from VWR International (Vienna, Austria); all had HPLC‐MS‐grade quality. Ultrapure water was prepared using an Arium 611 UV system (Sartorius, Göttingen, Germany). For the preparation of extracts, ultrapure water was used. Standards were isolated by one of the authors (A.O.) or available from previous projects. 4‐Deoxygadusol was provided by (I.Z.). Their purity (≥95%) and identity were always confirmed by UPLC‐UV, UPLC‐MS, and NMR; respective chromatograms/spectra are available upon request.

### 2.3 HILIC‐UPLC‐DAD‐MS/MS

HILIC‐UPLC‐DAD‐MS/MS experiments were conducted on an Acquity system from Waters (Milford, MA, USA) comprising a binary pump, autosampler, column oven, DAD, and a triple quadrupole mass spectrometer (Xevo TQD, Waters). Electrospray ionization (ESI) experiments were conducted with a ZSpray source in positive mode: capillary voltage +3.50 kV, cone voltage 50 V, collision energy 20 eV, desolvation temperature 600°C, and source temperature 150°C operated in multi reaction monitoring (MRM) mode. The instrument was operated by MassLynx software (V4.2). As stationary phase, a Triart Diol HILIC column (50 mm × 2.1 mm, 1.9 µm particle size; YMC, Dinslaken, Germany) was used. The mobile phase comprised ACN/water (9:1, v/v) containing 5 mM ammonium acetate, adjusted to pH 6.5 with acetic acid (A), and ACN/water (1:1, v/v) containing 15 mM ammonium acetate, adjusted to pH 3.5 with acetic acid (B). The gradient started with 0% B and changed in 10 min from 0% to 35% B. Thereafter, a washing step with 100% B for 10 min was performed. Finally, re‐equilibration for 10 min with initial conditions was applied. Flow rate, column temperature, and injection volume were set to 0.5 mL/min, 5°C, and 5 µL, respectively. Detection wavelengths were adjusted to 275 and 330 nm.

### 2.4 Calibration and Method Validation

The developed HILIC‐UPLC‐DAD‐MS/MS method was validated at research level according to ICH guidelines [18] to ensure fulfillment of current regulatory standards. Results of the method validation are shown in detail in Section 3.2.

#### 2.4.1 Linearity, Limit of Detection (LOD), and Limit of Quantification (LOQ)

Stock solutions of standards were prepared by dissolving the separately weighed substances in ACN/Water (9:1, v/v) at a concentration level of 250 µg/mL. A total of 15 calibration levels were prepared by serial dilution in the ratio 1:1 of each of them with ACN/Water (9:1, v/v). By plotting the measured peak area against concentrations, calibration curves were generated. Regression parameters were determined using Microsoft Excel. The LOD was expressed as 3.3 times the standard deviation of *y*‐intercept divided by slope; the LOQ corresponded to 10 times this value [18].

#### 2.4.2 Precision and Accuracy

Five individual sample solutions of *Porphyra* sp. (a) were prepared on each of three consecutive days following the above‐described extraction protocol. Intraday precision was evaluated on the basis of the results obtained on single days; for inter‐day consistency, the data of all 3 days were compared. Variations were calculated using peak areas and expressed as relative standard deviation (RSD). Accuracy was assessed by individually spiking the dry sample of *Fucus spiralis* with known amounts of standards at three concentration levels (1, 2.5, and 5 µg/mL, always related to the final sample volume of 25 mL) prior to sample extraction. The respective volumes were added to the accurately weighed biomass placed in a centrifuge tube. All solutions were prepared in triplicate, and after analysis, the theoretically present concentration was compared to the actually determined one; the recovery rate was expressed in percent.

## 3 Results and Discussion

### 3.1 Method Development

**FIGURE 1:**
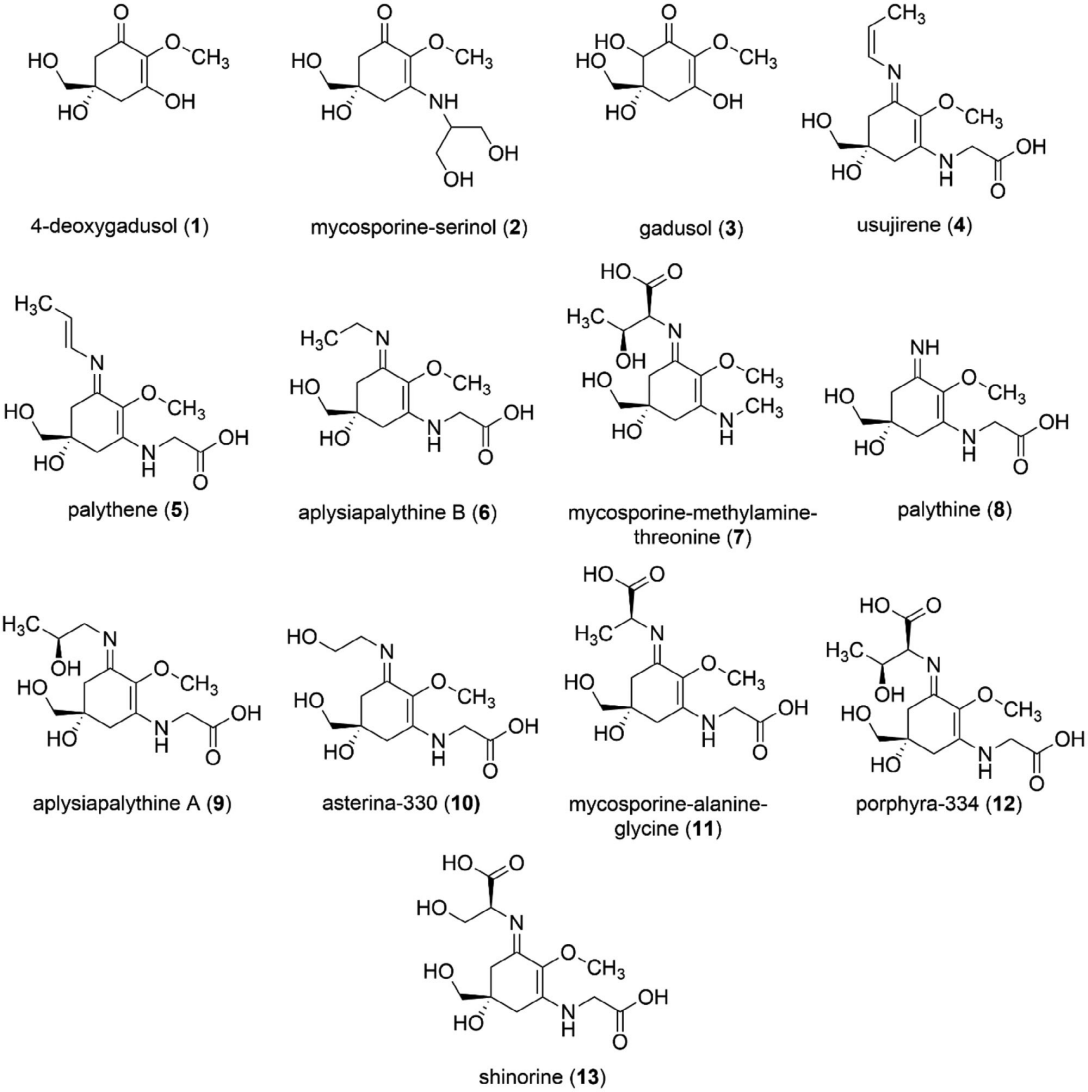
Chemical structure of the MAAs and their precursors 4‐deoxygadusol and gadusol, analyzed in this study.

For development of the HILIC‐UPLC‐DAD‐MS/MS method, a mixture of 11 MAAs and 2 of their precursors was prepared in ACN/water (9:1, v/v, 20 µg/mL; see Figure 1 for structures). Our investigation began with identifying an optimal stationary phase that would enable a rapid and efficient separation of all standard compounds. Due to their pronounced polarity, HILIC phases seem to be an excellent choice for the separation of MAAs, and two of them were tested: ACQUITY UPLC BEH HILIC from Waters and a YMC Triart‐Diol HILIC, both with identical dimensions (2.1 mm × 50 mm) and comparable particle size (1.7 and 1.9 µm). The latter consists of hybrid silica particles functionalized with 2,3‐dihydroxypropyl groups, being ideal for the separation of analytes containing hydroxy‐ and amino‐groups. According to the manufacturer, this material shows low nonspecific adsorption and can be used for the separation of small, polar molecules within a pH range of 2 and 10. These positive characteristics were not only observed in previous studies [19, 20, 21, 22], but also valid for the current one. For the separation of MAAs this phase revealed the best performance in terms of peak shape and overall separation efficiency, indicated by a peak resolution ≥2.3 (see Figure S1). Exceptions are the separation of mycosporine‐alanine‐glycine and porphyra‐334 (**11 + 12**), which show an *R*_s_‐value of 1.39, as well as porphyra‐334 and shinorine (**12 + 13**), which exhibit a peak resolution of 1.49. Only usujirene + palythene (**4 + 5**) and palythine + aplysiapalythine A (**8 + 9**) could not be separated. Regarding the former, this is due to the fact that they are not only *cis/trans*‐configurational isomers but also less hydrophilic and thus less retained on HILIC material. Their separation, which is possible on RP phases, might have been feasible in prolonged analysis time; however, this was not considered due to their rare occurrence in biological material [15, 23]. Quantifying them individually also adds little benefit as they form a palythene–usujirene mixture in the ratio 11:1 in aqueous solutions anyway [24]. Therefore, both substances were quantified together. Compounds **8** and **9**, on the other hand, differ by an additional 2‐hydroxypropyl sidechain, which apparently has no impact on retention under the applied conditions. Asterina‐330 (**10**), displaying a hydroxyethyl residue, instead interacts longer with the HILIC surface than **8** and **9**, resulting in a nicely resolved peak.

A gradient system comprising water and ACN is the first choice when developing a HILIC method for MAAs [11], even if the exact retention mechanism of this type of stationary phase is still not fully understood [25]. It is discussed that a combination of hydrophilic partitioning with the water‐rich layer at the stationary phase surface, adsorption, and electrostatic interactions, when charged species are involved, are relevant [26]. Thus, mobile phase composition, buffer concentration, pH, and temperature usually have a strong impact on the result [27]; however, gradient elution conditions make it difficult to define a single dominant retention process, because the structure of the adsorbed water layer and interaction mechanisms continuously change [28]. In the current case, the standard compounds could not be resolved with ACN/water (9:1, v/v, A) and ACN/water (1:1, v/v, B) as eluents, applying a linear gradient from 0% B to 35% B over 10 min (Figure S2B). In literature, the use of additives (e.g., ammonium formate, ammonium acetate, formic acid, or acetic acid) is suggested to enhance separation efficiency [7, 8]. Karatapanis et al. reported that also the buffer concentration is important in HILIC as it affects the type and extent of interaction between analytes and stationary phase [22]. Thus, the effects of ammonium acetate and ammonium formate, individually added at concentrations of 5–15 mM to both phases, were evaluated. As can be seen in Figure S2C, with ammonium formate the separation efficiency clearly improved. However, ammonium acetate showed to be even more advantageous in terms of peak shape, and a higher buffer molarity was beneficial for the resolution of later eluting standards. As also the pH value is a critical factor to consider during method development, both mobile phases were set to the same pH first (6.5, 5.5, 4.5, and 3.5; values adjusted with acetic acid). It was noticed that a higher pH (6.5) was advantageous for the separation of earlier eluting MAAs, whereas at pH 3.5 **11**–**13** could be better resolved. These results indicated the optimal mobile phase comprises ACN/water (9:1, v/v) with 15 mM ammonium acetate, pH 6.5 as solvent A and ACN/water (1:1, v/v) with 15 mM ammonium acetate, pH 3.5 as solvent B.

Naturally, separation temperature and mobile phase gradient have an impact on HILIC separations as well [26]. This is clearly indicated when comparing the separation of MAAs at 5°C and 15°C. The former enables a much better peak resolution, especially between the analytes **11**–**13** (see Figure S3B). Regarding the optimal gradient, with a linear change from 0% to 100% solvent B in 10 min, the target analytes eluted too quickly and were not well resolved; a gradient from 0% to 25% B in 10 min resulted in a much longer separation time without obvious benefits (e.g., Compounds **8** and **9** still coeluted). These results indicated that the optimal gradient had to be a compromise between both options. Therefore, a gradient from 0% to 35% solvent B in 10 min was selected. It permitted the overall best separation of standards **1**–**13** in just below 5 min (see Figure S3C,D).

**FIGURE 2:**
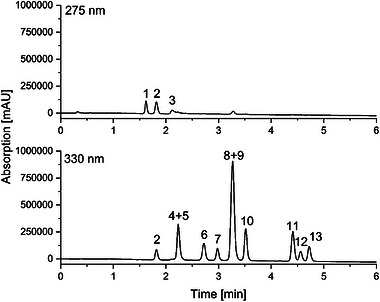
HILIC‐UPLC‐DAD separation of standard compounds under optimized conditions. Stationary phase: YMC Triart Diol HILIC column (50 mm × 2.1 mm, 1.9 µm particle size); mobile phase: ACN/water (9:1, v/v) containing 5 mM ammonium acetate, adjusted to pH 6.5 with acetic acid (A), ACN/water (1:1, v/v) containing 15 mM ammonium acetate, adjusted to pH 3.5 with acetic acid (B); gradient: in 10 min from 0% to 35% B; wash: 10 min with 100% B; re‐equilibration: 10 min with initial conditions; flow rate: 0.5 mL/min; column temperature: 5°C; sample volume: 5 µL. Compounds: 4‐deoxygadusol (**1**), mycosporine‐serinol (**2**), gadusol (**3**), usujirene + palythene (**4 + 5**), aplysiapalythine B (**6**), mycosporine‐methylamine‐threonine (**7**), palythine + aplysiapalythine A (**8 + 9**), asterina‐330 (**10**), mycosporine‐glycine‐alanine (**11**), porphyra‐334 (**12**), and shinorine (**13**).

The flow rate was set to 0.5 mL/min in order to achieve maximum peak resolution while maintaining a short analysis time. When using HILIC, re‐equilibration is another critical factor. If not adequately performed, poor reproducibility is the consequence. For this application this was not a problem if a 10 min washing step (just required in sample analysis) with 100% B was followed by 10 min re‐equilibration under the initial conditions. This resulted in a total runtime of 30 min per sample. An injection volume of 5 µL assured adequate sensitivity. Dissolving the standards in mobile phase A (ACN/water, 9:1, v/v) without additives is important to ensure the reproducibility of results. Notably, the precursor compounds 4‐deoxygadusol (**1**) and gadusol (**3**) are only/better detectable at lower wavelengths (275 nm), whereas all other standards are well assignable at 330 nm (Figure 2). Therefore, DAD detection was set to 275 and 330 nm, based on the absorption maxima of the standard compounds determined during preliminary trials and according to literature [15, 23].

Figure 2 shows the separation of all 13 standards under optimized HILIC‐UPLC conditions, recorded at 275 and 330 nm. At 275 nm, the MAA precursors 4‐deoxygadusol (**1**) and gadusol (**3**), as well as the MAA mycosporine‐serinol (**2**), are clearly visible, whereas all naturally occurring MAAs in the mixture exhibit strong absorption at 330 nm.

LC–MS/MS experiments were conducted not only to assure peak identity but also to quantify the two co‐eluting MAAs palythine (**8**) and aplysiapalythine A (**9**). Respective data were recorded in positive ESI mode, and to maximize specificity, MRM was selected. For palythine, with retention time 3.46 min, the parent ion was 245.10 (*m/z*) and the daughter ion 209.02 (*m/z*). For aplysiapalythine A, with retention time 3.43 min, they were 303.20 (*m/z*) and 288.24 (*m/z*), respectively (see Figure S4).

**FIGURE 3:**
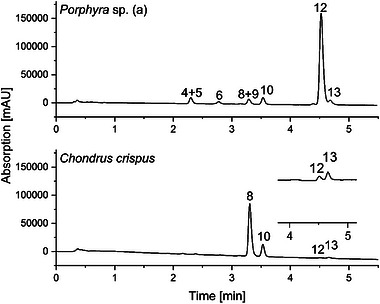
HILIC‐UPLC‐DAD separation of authentic algal extracts under optimized conditions: *Porphyra* sp. (a) and *Chondrus crispus*. The detection wavelength was set to 330 nm. Separation conditions and peak assignments can be found in the caption of Figure 2.

### 3.2 Method Validation

By conducting validation experiments according to ICH guidelines, the suitability of the developed method for the quantitative evaluation of MAAs and respective precursors in real samples, that is, algal extracts, was confirmed. In Tables 1, 2, 3 the obtained results are summarized.

**TABLE 1:** Calibration data for selected compounds determined at 330 nm, except for Compounds **1** and **3**, which were both quantified using a wavelength of 275 nm; **4** and **5** were considered a single compound, as they constitute a *cis/trans* isomeric pair capable of interconversion; **8** and **9** were determined by LC–MS in MRM mode.

Calibration data					
Compound	Regression equations	*R* ^2^	Linear range (µg/mL)	LOD (µg/mL)	LOQ (µg/mL)
1	*Y* = 253.6*x* + 29.0	0.9999	0.12–250	0.030	0.100
2	*y* = 1314.6*x* + 28.1	0.9999	0.01–62.50	0.003	0.010
3	*y* = 301.6*x* − 928.3	0.9969	0.98–125	0.084	0.256
4 + 5	*y* = 1508.1*x* − 23.6	0.9999	0.06–62.50	0.010	0.031
8 MRM	*y* = 20.5*x* − 2.7	0.9959	0.49–250	1.08 × 10^−6^	3.27 × 10^−6^
9 MRM	*y* = 2228.8*x* − 556.0	0.9961	0.01–250	1.99 × 10^−3^	6.02 × 10^−3^
10	*y* = 723.0*x* − 91.5	0.9999	0.06–125	0.014	0.041
12	*y* = 1220.7*x* − 166.0	0.9999	0.06–125	0.015	0.049
13	*y* = 319.8*x* − 19.2	0.9999	0.06–62.50	0.015	0.045

Abbreviations: LOD, limit of detection; LOQ, Limit of Quantification; MRM, multi reaction monitoring.

**TABLE 2:** Precision of the developed assay based on mg mycosporine‐like amino acid (MAA) per g dry weight (DW) biomaterial.

Compound	Relative standard deviation			
	Day 1	Day 2	Day 3	Inter‐day
**8**	7.30	5.47	9.84	1.52
**9**	2.22	2.91	3.69	2.42
**10**	2.65	4.84	2.13	1.27
**12**	5.52	7.65	4.08	6.19
**13**	4.60	8.47	8.35	9.19

*Note*: Intraday (*n* = 5) and inter‐day (*n* = 3) variation in percent was determined using *Porphyra* sp. (a), extracted and analyzed as described in Section 2.1.

**TABLE 3:** Accuracy of the developed method.

Compound	Recovery rate (%)		
	Low	Medium	High
**1**	94.38 (4.64)	94.27 (3.08)	108.26 (1.78)
**2**	97.82 (2.08)	106.93 (1.68)	108.44 (0.38)
**8**	99.35 (7.06)	95.58 (2.69)	100.38 (5.77)
**9**	99.86 (4.14)	102.15 (6.16)	105.05 (9.42)
**10**	95.10 (5.43)	107.29 (1.10)	101.80 (0.24)
**12**	102.89 (3.84)	98.34 (1.98)	97.88 (1.14)

*Note*: Recovery rates at three spiking levels were determined using *Fucus spiralis*, extracted and analyzed as described in Section 2.1. Results are reported as mean values (*n* = 3) with corresponding relative standard deviation in parentheses.

Selectivity of the method was ensured by the absence of partially co‐eluting signals (shoulders) and highly consistent UV spectra across the relevant peaks (determined by using the peak purity function in the operating software), as well as LC–MS/MS data, which agreed to literature. For compounds available in sufficient amount (**1**–**5**, **8**–**10**, **12**, and **13),** calibration curves could be generated (Table 1). The linear range was determined from 0.01 to 250 µg/mL (only concentration levels confirmed to be within the detector's linear range were included), and the coefficient of determination (*R* ^2^) was found to be 0.9959 or higher. Compounds **6**, **7**, and **11** were only used as standards during method development but not quantified due to limited availability. The two co‐eluting MAAs palythine (**8**) and aplysiapalythine A (**9**) were quantified individually using MRM. Selecting this mode increases analytical selectivity and sensitivity [29]. This raises the question why not all analytes were determined accordingly. MAAs show a particularly strong UV absorption, rendering the use of MS for sensitive detection not essential, unless peaks coelute. Using UV‐DAD is a much more practical and cost‐efficient alternative instead. The two MAAs, usujirene (**4**) and palythene (**5**), mostly occurring in minor concentrations, are *cis/trans* configurational isomers, and even by MRM they cannot be differentiated; their UV maximum is slightly different (usujirene: 359 nm, palythene: 355 nm). This, however, was not sufficient for their distinct determination, and thus both were quantified together. Even if the two MAAs could be successfully resolved, they would undergo re‐isomerization in solution, yielding the geometric equilibrium mixture of usujirene (**4**) and palythene (**5**) [24].

When using UV‐detection, the calculated values for LOD and LOQ were always ≤0.08 and ≤0.26 µg/mL, respectively. Regarding precision, the highest intraday deviation was 9.84% (corresponding to Compound **8** on Day 3) and the variance between three consecutive days was always ≤9.19% (corresponding to Compound **13**), as presented in Table 2. Recovery rates were in the range of −5.73% (94.27%) (corresponding to Compound **1**, medium spike) to +8.44% (108.44%) (corresponding to Compound **2**, high spike) as seen in Table 3. Accordingly, all validation parameters were within acceptable ranges, and thus the method is suitable for practical application.

### 3.3 HILIC‐UPLC‐DAD‐MS/MS Analysis of MAAs in Different Algal Species

As proof of concept, algal samples representing different genera and geographical origins were analyzed using the newly developed method (see Figure 3 and Table 4). The MAAs asterina‐330 (**10**), porphyra‐334 (**12**), and shinorine (**13**) were found in almost all of the analyzed specimens. In contrast, palythine (**8**) and aplysiapalythine A (**9**) were detected only in approximately half of them. Concerning the latter two compounds, well acceptable values for accuracy and precision indicate that they can be quantified in MRM mode with confidence even without using an internal standard (besides, deuterated MAAs are not available). Unlike mycosporine‐serinol (**2**) and the MAA precursors 4‐deoxygadusol (**1**) and gadusol (**3**), which could never be confirmed. Usujirene (**4**) and palythene (**5**), although not quantified individually, were consistently detected in all *Porphyra* sp. (a–d) samples. The MAA profiles exhibited substantial qualitative and quantitative variability across species. For example, the two brown algae *F. spiralis* and *Himanthalia elongata* did not contain any of the MAAs investigated in this study. In contrast, *Porphyra* sp. (a–d) consistently contained at least five of the MAAs analyzed. Notably, qualitative and quantitative differences were observed among the commercially obtained *Porphyra* sp. samples (a–d), which may be explained by seasonal variations and different growth locations (see Table S2). The red alga *V. lanosa* showed the highest abundance of the MAA aplysiapalythine A (**9**). Porphyra‐334 (**12**) and shinorine (**13**) dominated in *Porphyra* sp. (a–d) samples. *Chondrus crispus* displayed the highest abundance of the MAA asterina‐330 (**10**), whereas the highest levels of the MAA palythine (**8**) were observed in the red alga *Grateloupia turuturu*.

**TABLE 4:** Quantitative mycosporine‐like amino acid (MAA) profiles in different algae as determined by hydrophilic interaction liquid chromatography (HILIC)‐ultra‐performance liquid chromatography (UPLC)‐diode array detector (DAD)‐tandem mass spectrometry (MS/MS) analysis.

Alga	Compound								
	1	2	3	4 + 5	8	9	10	12	13
*Calliblepharis jubata*	—	—	—	—	0.07 (7.77)	—	0.06 (1.21)	0.05 (0.71)	—
*Ceramium* sp.	—	—	—	—	0.14 (3.91)	—	Det.	Det.	Det.
*Chondrus crispus*	—	—	—	—	0.26 (2.24)	—	0.51 (0.89)	0.06 (1.35)	0.18 (2.44)
*Cladophora* sp.	—	—	—	—	—	—	—	0.05 (3.18)	—
*Fucus spiralis*	—	—	—	—	—	—	—	—	—
*Gracilaria gracilis*	—	—	—	—	—	—	Det.	0.07 (0.40)	0.33 (0.54)
*Grateloupia turuturu*	—	—	—	—	0.40 (4.99)	—	0.06 (3.13)	Det.	Det.
*Himanthalia elongata*	—	—	—	—	—	—	—	—	—
*Jania rubens (a)*	—	—	—	—	0.08 (6.67)	—	Det.	0.07 (2.08)	Det.
*Jania rubens (b)*	—	—	—	—	0.13 (8.27)	—	Det.	0.08 (0.50)	Det.
*Lomentaria articulata*	—	—	—	—	—	—	Det.	Det.	—
*Mastocarpus stellatus*	—	—	—	—	—	—	0.05 (0.84)	0.07 (0.92)	0.17 (0.70)
*Osmundea* sp.	—	—	—	—	0.11 (8.39)	0.09 (1.48)	0.09 (0.92)	0.08 (0.67)	Det.
*Pelvetia canaliculata*	—	—	—	—	0.06 (9.30)	—	—	0.12 (0.93)	—
*Porphyra* sp. *(a)*	—	—	—	Det.	0.22 (2.56)	0.07 (0.39)	0.30 (3.98)	2.96 (0.88)	0.88 (1.10)
*Porphyra* sp. *(b)*	—	—	—	Det.	—	0.07 (0.91)	0.14 (1.23)	1.88 (0.08)	1.94 (0.42)
*Porphyra* sp. *(c)*	—	—	—	Det.	0.15 (3.91)	0.07 (0.32)	0.31 (0.61)	1.94 (0.19)	0.28 (2.35)
*Porphyra* sp. *(d)*	—	—	—	Det.	—	0.06 (4.91)	0.32 (0.05)	1.89 (0.01)	0.32 (0.86)
*Saccharina latissima*	—	—	—	—	—	—	—	—	Det.
*Ulva lactuca*	—	—	—	—	—	—	—	—	Det.
*Vertebrata lanosa*	—	—	—	—	0.37 (2.66)	0.21 (2.37)	0.05 (2.48)	0.09 (0.67)	Det.

*Note*: Det. = compound detected but not quantified as the concentration was below the LOQ. Mean values expressed as mg per g of dry weight (DW) biomaterial with corresponding relative standard deviation in parentheses. Assignment of compounds as in Figure 1.

It should be emphasized that the primary objective of this study was the optimization, validation, and practical application of HILIC‐UPLC for MAA analysis in algae rather than a chemosystematic study. For such an investigation the number of assayed samples per species was much too small, not allowing any general conclusions. However, the results are comparable with those of previous studies, using other analytical methods. For example, in the RP‐UHPLC study by Zwerger et al., three different *Porphyra* sp. samples were analyzed, and similar qualitative and quantitative MAA patterns were reported. For example, the samples analyzed in our study contained porphyra‐334 at 1.88–2.96 mg/g, shinorine at 0.28–1.94 mg/g, asterina‐330 at 0.14–0.32 mg/g, and palythine at 0.06–0.07 mg/g dry weight (DW) algae. Concerning the same species, Zwerger et al. reported comparable concentrations for porphyra‐334 (4.36 mg/g DW algae), shinorine (0.21 mg/g DW algae), asterina‐330 (0.27 mg/g DW algae), and palythine (0.13 mg/g DW algae) [15]. Overall, there is a good agreement, especially considering that the samples were not identical and others were reanalyzed after more than 3 years of storage. Several other previous studies only analyzed the overall MAA content in mg/g DW biomaterial and did not quantify MAAs individually [30, 31].

## 4 Conclusion

A novel HILIC‐UPLC method was developed for the analysis of eleven MAAs as well as two of their precursors, and finally, 11 of them could be separated chromatographically. For the first time, method development and validation were performed not only considering naturally occurring MAAs, but also the biosynthetic precursors of MAAs. Even if 4‐deoxygadusol (**1**) and gadusol (**3**) could not be detected in any of the analyzed samples, this aspect could be of interest for further studies on the biosynthesis of these highly interesting natural products, as well as for monitoring intermediate products during the chemical synthesis of MAAs. The problem of the co‐eluting compounds palythine (**8**) and aplysiapalythine A (**9**) was resolved by their specific determination using MRM. Only the two isomers, usujirene (**4**) and palythene (**5**), had to be quantified together, but they are usually less relevant due to their low occurrence in algae. Even so, none of the previously reported analytical methods was able to resolve that many MAAs in such a short analysis time of less than 5 min. This speed has no negative impact on the performance characteristics of the assay (as confirmed by method validation) or reliability of results (as shown by a good agreement of quantitative results with those of previous studies). This method also represents an orthogonal chromatographic strategy to the much better‐established RP‐(U)HPLC assays. Orthogonality is required by ICH to confirm validation parameters like selectivity and accuracy, and of general importance for the quantification of active pharmaceutical ingredients (APIs) in products. If the results of two orthogonal approaches match well, the true value in a product is confirmed [32].

MAAs are natural products of considerable interest for the cosmeceutical industry, primarily due to their strong UV‐absorbing properties and high extinction coefficients. The developed approach is relevant not only because of its innovation in terms of the described analytical setup (HILIC in combination with UPLC) but also due to its practical relevance. For that reason, the non‐co‐eluting MAAs were quantified with a DAD, which is the most commonly used detector. For the few derivatives that could not be chromatographically resolved, the use of LC–MS/MS proved to be an option, both in respect to a conclusive assurance of identity and quantity. The high separation efficiency of the assay was not limited to the separation of standards alone but could be transferred to authentic, complex algal extracts as well. Accordingly, the presented method creates novel opportunities in MAA research, further extending the limits of separation speed and efficiency.

## Author Contributions

**Armin Oberosler**: investigation, methodology, conceptualization, data curation, formal analysis, validation, visualization, writing – original draft. **Anastasiia Fedorova**: investigation. **Ignacio Zegri**: resources (provision of chemically synthesized 4‐Deoxygadusol (**1**). **Fabian Hammerle**: methodology, supervision, conceptualization, writing – review and editing. **Michael Zwerger**: methodology, supervision, conceptualization, writing – review and editing. **Thomas Werner**: methodology, supervision, conceptualization. **Markus Ganzera**: conceptualization, supervision, writing – review and editing, project administration, funding acquisition.

[Correction added on June 19 2026, after first online publication: contribution from the sixth author Thomas Wernerin is added in this version.]

## Funding

This research was funded in part by the Austrian Science Fund (FWF) [Grant‐DOI: 10.55776/I6122, UVISION 1.1]. The German Science Foundation (DFG) is gratefully acknowledged for financial support (WE 3605/6‐1). For open access purposes, the author has applied a CC BY public copyright license to any author accepted manuscript version arising from this submission.

## Conflicts of Interest

The authors declare no conflicts of interest.

## Supporting information




**Supporting File**: elps70110‐sup‐0001‐SuppMat.docx.

## Data Availability

The data that support the findings of this study are available from the corresponding author upon reasonable request.
