# Supplementary material for: A Novel HILIC‐UPLC‐DAD‐MS/MS Method for the Analysis of Mycosporine‐Like Amino Acids and Their Quantification in Diverse Algae
Source: Electrophoresis. 2026 May 23;47(7):620–8. doi: 10.1002/elps.70110 (PMC13378235; doi:10.1002/elps.70110)
Supplement: Supplementary file 1 — Supporting File: elps70110‐sup‐0001‐SuppMat.docx. [file ELPS-47--s001.docx]

**A novel HILIC-UPLC-DAD-MS/MS method for the analysis of mycosporine-like amino acids and their quantification in diverse algae**

Supplementary information

Armin Oberosler^1^, Anastasiia Fedorova^1^, Ignacio Zegri^3^, Fabian Hammerle^1^, Michael Zwerger^2^, Markus Ganzera^1*^

^1^Institute of Pharmacy, Pharmacognosy, University of Innsbruck, Innsbruck, Austria;

^2^Institute of Medical Biochemistry, Medical University of Innsbruck, Innsbruck, Austria;

^3^University of Paderborn, Paderborn, Germany

*Corresponding author:

Assoc.-Prof. Dr. Markus Ganzera

Institute of Pharmacy, Pharmacognosy, University of Innsbruck

Innrain 80-82, 6020 Innsbruck, Austria

# HILIC-UHPLC-DAD-MS method development

**Table S1**. Overview of tested stationary phases

| Manufacturer | Stationary phase | Dimensions |
| --- | --- | --- |
| Waters Corporation | ACQUITY UPLC BEH HILIC | (2.1 mm x 50 mm; 1.7 µm) |
| YMC Company, Limited | YMC-Triart Diol-HILIC | (2.1 mm x 50 mm; 1.9 µm) |


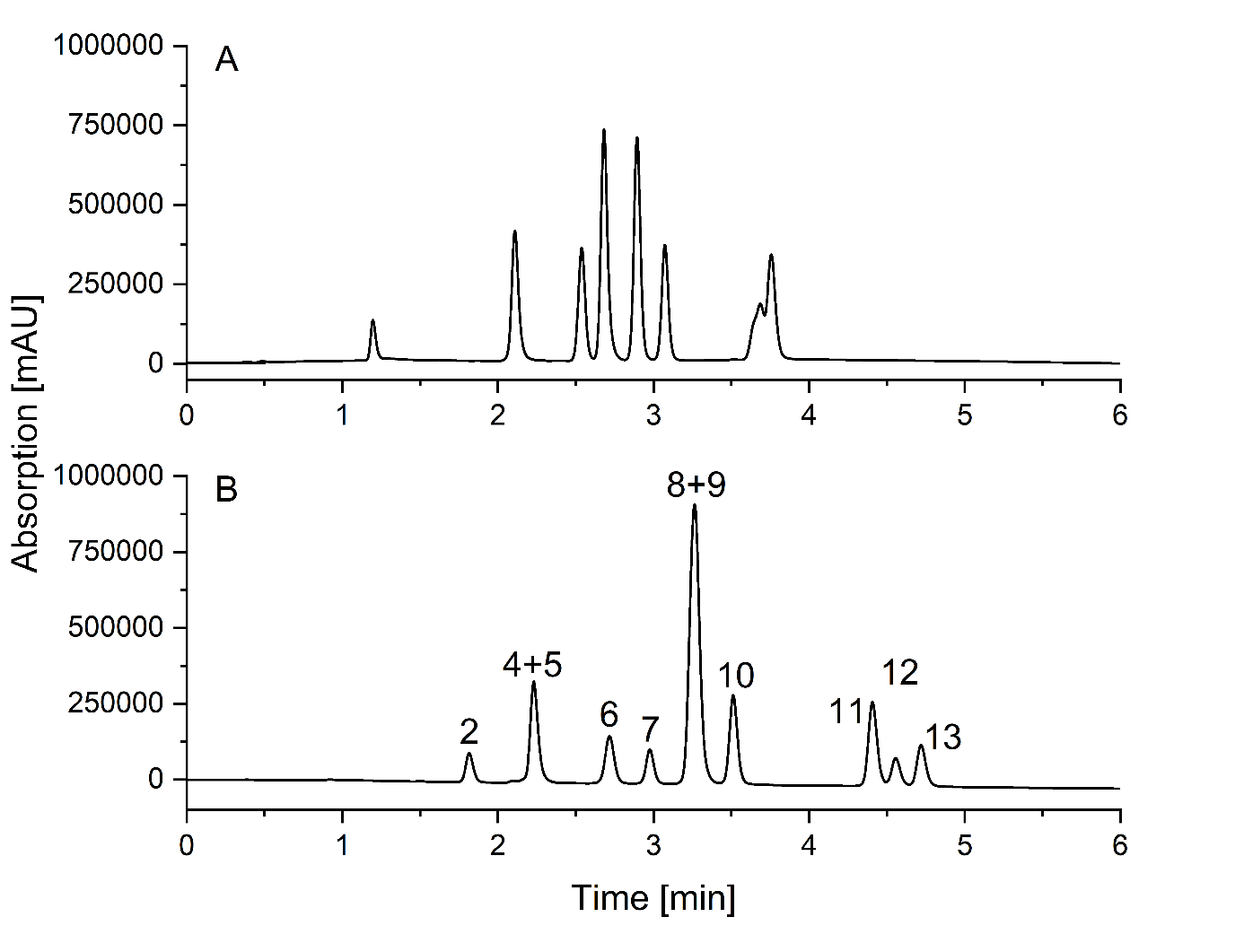


**Figure S1**. Comparison of the two stationary phases based on the analysis of a standard mix (20 µg/mL in ACN/water (9:1, v/v)) monitored at 330 nm. (**A**): Acquity UPLC BEH HILIC (2.1 mm × 50 mm; 1.7 µm); (**B**): YMC-Triart Diol-HILIC (2.1 mm × 50 mm; 1.9 µm). All other conditions were optimal. 4-Deoxygadusol (**1**) and gadusol (**3**), mycosporine-serinol (**2**), usujirene (**4**), palythene (**5**), aplysiapalythine B (**6**), mycosporine-methylamine-threonine (**7**), palythine (**8**), aplysiapalythine A (**9**), asterina-330 (**10**), mycosporine-alanine-glycine (**11**), porphyra-334 (**12**), and shinorine (**13**).


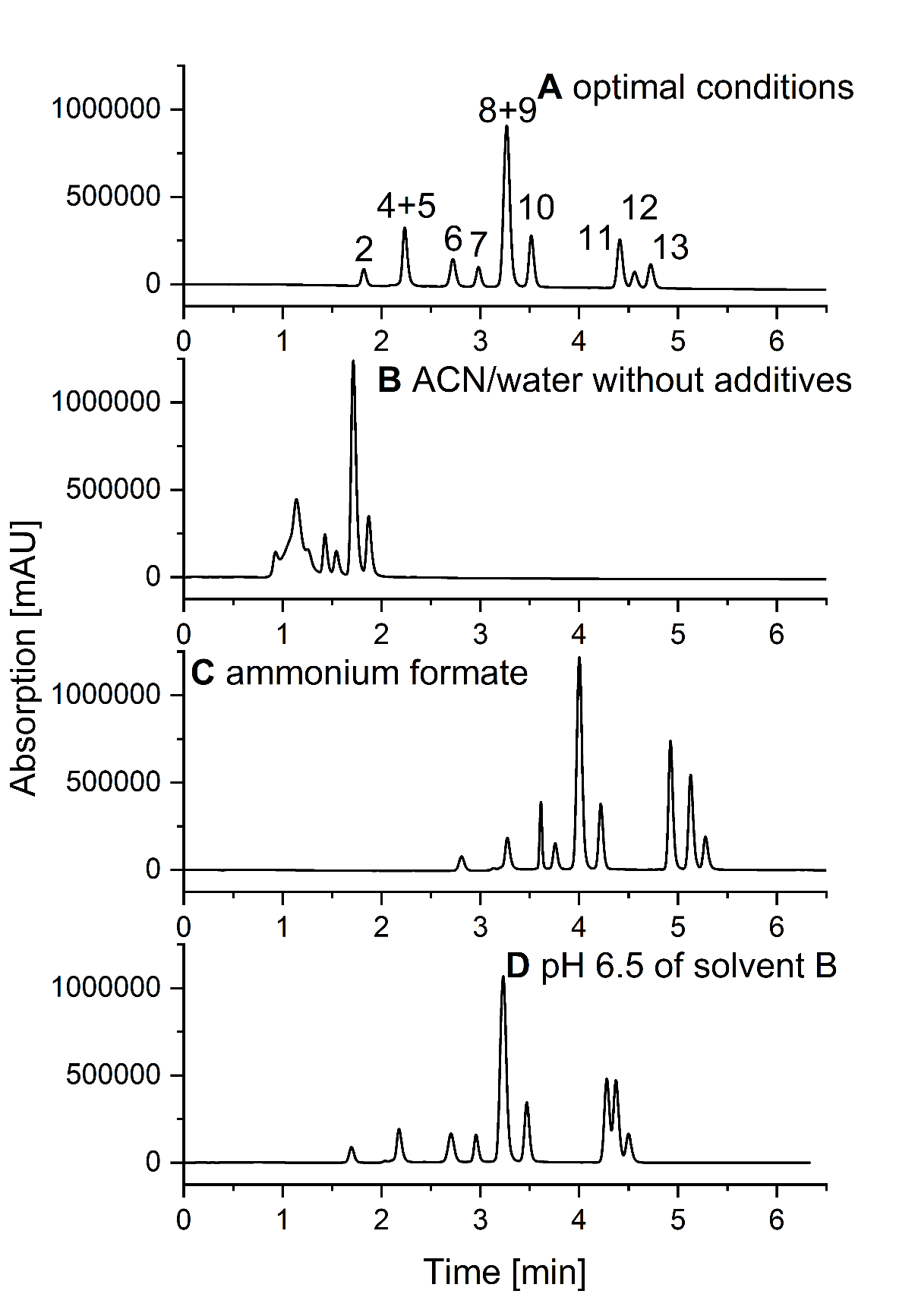


**Figure S2**. Influence of different mobile phase compositions: Optimum (**A**), ACN/water (9:1, v/v) without additives and ACN/water (1:1, v/v) without additives (**B**), ACN/water (9:1, v/v) with 5 mM ammonium formate pH 6.50 and ACN/water (1:1, v/v) with 15 mM ammonium formate pH 3.50 (**C**), ACN/water (9:1, v/v) with 5 mM ammonium acetate pH 6.50 and ACN/water (1:1, v/v) with 15 mM ammonium acetate pH 6.50 (**D**). All other settings were optimal.


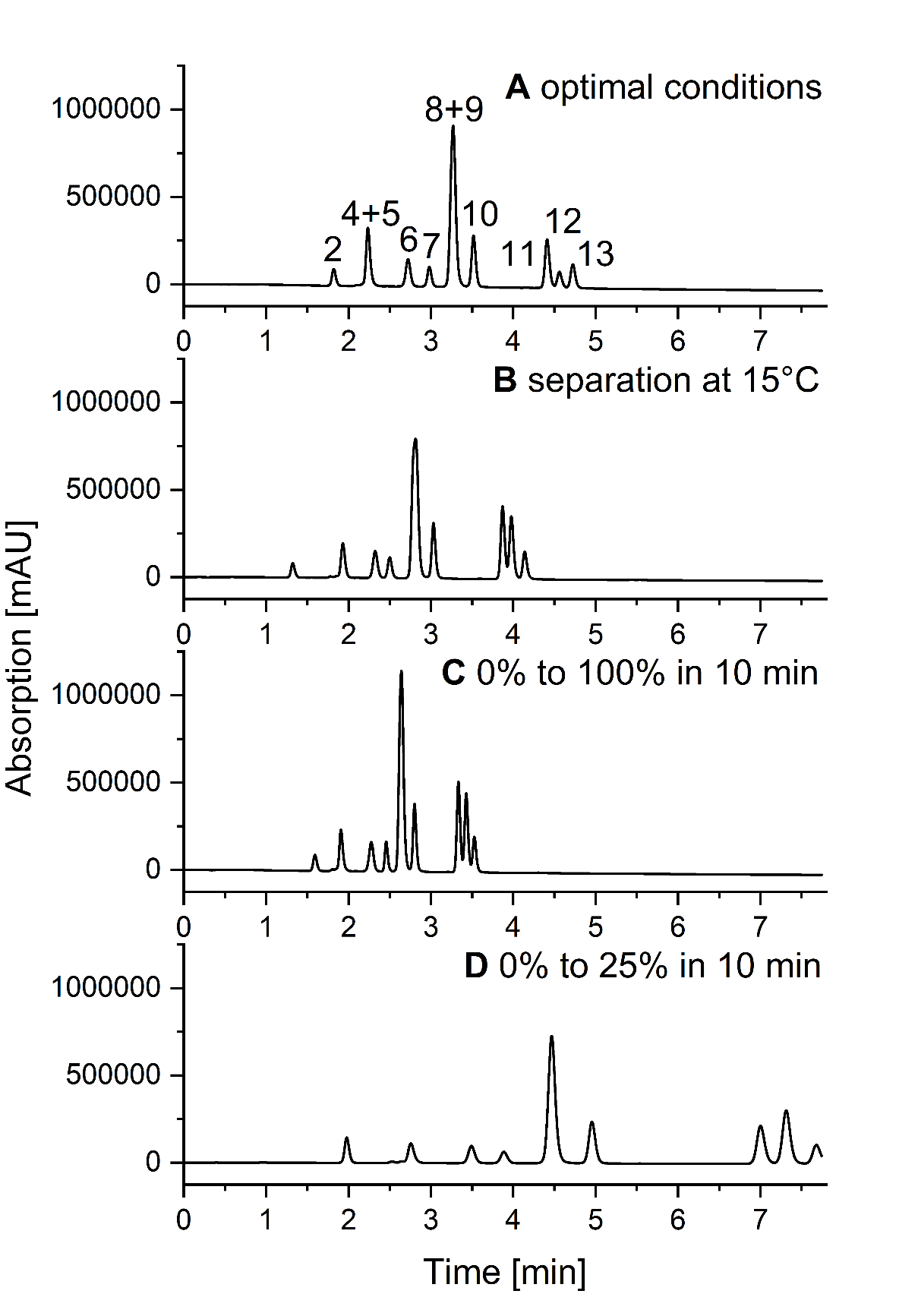


**Figure S3**. Influence of temperature and gradient on the separation of eleven MAA standards and two of their precursors, 4-deoxygadusol and gadusol: Optimal conditions (**A**), separation at 15 °C (**B**), gradient from 0 to 100% B in 10 min (**C**), gradient from 0 to 25% B in 10 min (**D**). All other settings were optimal.

# Analyzed algal samples

**Table S2**. Provenance and taxa of the anlyzed algae.

| Species | Family,  Order | Collection Place | Country | Collection Date |
| --- | --- | --- | --- | --- |
| *Calliblepharis jubata* | Cystocloniaceae, Gigartinales | Roscoff | France | 6/2018 |
| *Ceramium* sp. | Ceramiaceae, Ceramiales | Roscoff | France | 6/2018 |
| *Chondrus crispus* | Gigartinaceae, Gigartinales | Roscoff | France | 6/2018 |
| *Cladophora* sp. | Cladophoraceae,  Cladophorales | Roscoff | France | 6/2018 |
| *Fucus spiralis* | Fucaceae,  Fucales | Roscoff | France | 6/2018 |
| *Gracilaria gracilis* | Gracilariaceae, Gracilariales | Roscoff | France | 6/2018 |
| *Grateloupia turuturu* | Halymeniaceae, Halymeniales | Roscoff | France | 6/2018 |
| *Himanthalia elongata* | Himanthaliaceae,  Fucales | Roscoff | France | 6/2018 |
| *Jania rubens* (a) | Corallinaceae, Corallinales | Roscoff | France | 6/2018 |
| *Jania rubens* (b) | Corallinaceae, Corallinales | Crete | Greece | 4/2018 |
| *Lomentaria articulata* | Lomentariaceae,  Rhodymeniales | Roscoff | France | 6/2018 |
| *Mastocarpus stellatus* | Phyllophoraceae, Gigartinales | Roscoff | France | 6/2018 |
| *Osmundea* sp. | Rhodomelaceae,  Ceramiales | Roscoff | France | 6/2018 |
| *Pelvetia canaliculata* | Fucaceae,  Fucales | Roscoff | France | 6/2018 |
| *Porphyra* sp. (a) | Bangiaceae, Bangiales | Not known* | China | Not known* |
| *Porphyra* sp. (b) | Bangiaceae, Bangiales | Not known* | Japan | Not known* |
| *Porphyra* sp. (c) | Bangiaceae, Bangiales | Not known* | South Korea | Not known* |
| *Porphyra* sp. (d) | Bangiaceae, Bangiales | Not known* | South Korea | Not known* |
| *Saccharina latissima* | Laminariaceae,  Laminariales | Roscoff | France | 6/2018 |
| *Ulva lactuca* | Ulvaceae,  Ulvales | Roscoff | France | 6/2018 |
| *Vertebrata lanosa* | Rhodomelaceae,  Ceramiales | Roscoff | France | 6/2018 |

* commercial sample

# MRM-Chromatogramm (LC-MS/MS; TQ)


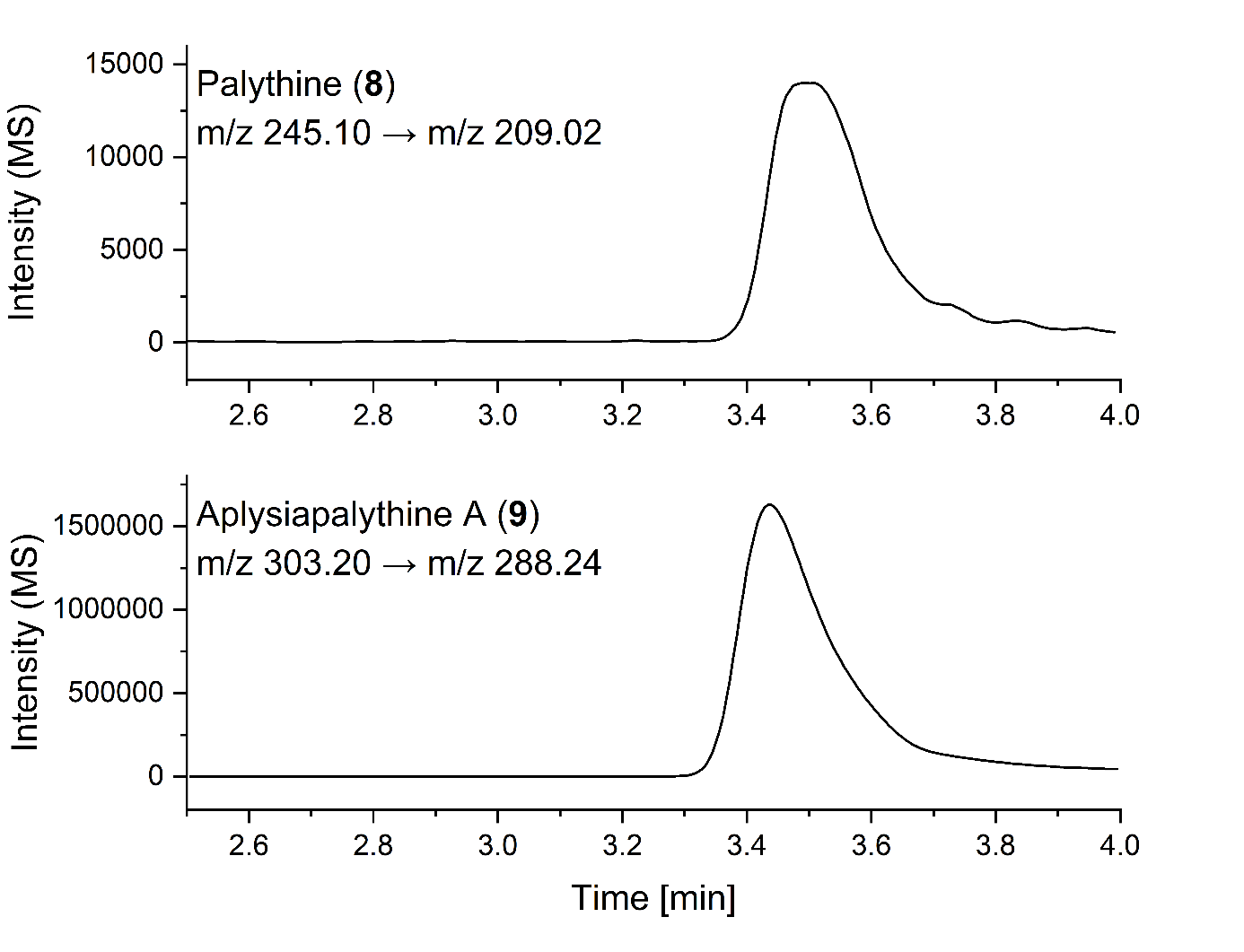


**Figure S4**. MRM transition of palythine (**8**) and aplysiapalythine A (**9**), data recorded in positive electrospray ionization mode. For **8**, the retention time was 3.46 min; for **9** it was 3.43 min.
